# Development of Preschool Children’s Executive Functions throughout a Play-Based Learning Approach That Embeds Science Concepts

**DOI:** 10.3390/ijerph18020588

**Published:** 2021-01-12

**Authors:** Clara Vidal Carulla, Nikolaos Christodoulakis, Karina Adbo

**Affiliations:** Department of Biology and Environmental Science, Linnaeus University, 39231 Kalmar, Sweden; clara.vidalcarulla@lnu.se (C.V.C.); nikolaos.christodoulakis@lnu.se (N.C.)

**Keywords:** science activities, chemistry, preschool, executive functions

## Abstract

This study focuses on the development of executive functions in preschool children during a series of science activities. A longitudinal play-based learning intervention was designed and implemented following the design of an educational experiment. Data were collected through visual ethnography in hot situations with adult supervision. Results show how entwined the concepts of inhibitory control and cognitive flexibility are within young children’s development. The development of cognitive flexibility or attention shifting readily occurred when there were fictive characters (such as the king and his royal family), but changing perspective toward a nonfictive environment (i.e., taking other children’s perspectives) was a more difficult and time-consuming process. This process began in an individual perspective and expanded to acknowledging others’ perspectives, then moved toward creating common perspectives or alternative narratives. Results show that science activities can be a bridge for preschool children to transfer their use of executive functions, from fairytales and games toward everyday tasks.

## 1. Introduction

There has been an increase in the number of publications concerning preschool science education during the past decades [1,2,3,4,5,6,7,8,9,10,11]. Much of the research efforts for this educational level have been focused on broadening children’s scientific experiences derived from the living, visual, everyday world, especially within the fields of biology [12,13,14,15] and physics [16,17,18,19]. However, very little is known about the processes influencing emergent science, such as what effect the development of self-regulation and executive functions has on science activities.

### 1.1. Introducing Science to Preschool Children

The use of the word “emergence” here holds a dual meaning; first, it suggests that the phenomena studied emerge, i.e., are new to the child and not things that the child has previous experience of; secondly, emergence places focus on the actual process of this development. To study emergent science also means that it is the child’s perspective of a scientific content that is the focus of the study. Emergence of abstract concepts is interdependent with several different processes that are nonlinear, dynamic, and intertwined with all of the different influences from the child’s everyday life. Indeed, social and emotional development is vital for children’s learning in all different environments, not least for “school readiness” [20].

One of the most wide-ranging perspectives of social and emotional development is a perspective that includes the aspects beyond cognitive development, namely, the ability to adjust intentionally to a changing external environment in order to achieve a specific purpose. This particular ability is deemed especially important for children to have when entering school. Executive functions (EFs) are a well-accepted term used for addressing these abilities. The research literature is in agreement that EFs are domain-general and thereby affecting all kinds of learning [21,22]; however, when viewed from the different perspectives within science literature, the definition of what is included within EFs varies. Indeed, Griffin et al. (2016) conclude that 100 researchers will have 100 different definitions of what is included within the concept. This situation is viewed as positive, as it promotes the development of the field rather than delimiting it. In the research literature, EFs have been referred to as “a family of skills” [23] or “a number of cognitive processes” [24] that support children in regulating their behavior, something that in turn is beneficial for social, emotional, and cognitive development.

Executive functions seem to be a combination of three overarching processes that have been referred to as cognitive control skills [22] or core executive functioning skills [21]. Three key processes are included. Working memory (WM), which is essential in order to remember instructions and be able to process information actively [25]. Inhibitory control (IC) is also important, especially for persistence, as it helps us to manage interferences, and the third and final control skill is attention shifting (AS) [21] which allows us to switch our attention and behavior in order to achieve an objective [26]. These functions are considered of higher order, because they include the voluntary and conscious control of thoughts, emotions, and behaviors [27]. From a developmental perspective, IC and WM are seen as interrelated; one of the arguments for this is that you need to remember what to focus on to be able to inhibit unrelated things. Certainly, for very young children, IC and WM seem to be developmentally undifferentiated. Attention shifting (AS), on the other hand, requires both WM and IC, and is viewed as something that may begin to emerge somewhat later on [28]. Here, resolving conflict is seen as one of the indicators of AS, i.e., a shift in attention set [29].

The different definitions of EFs and other related concepts also depend, of course, on the perspective chosen for describing EFs. The descriptions may also be made from metacognition, i.e., they are already developed functions in an adult, or they are seen as developing and then derived from children’s development.

### 1.2. Self-Regulation Skills from an Adult Metacognition Perspective

Another way to describe these skills can be by using the concept of self-regulation. This concept can be defined by taking the adult perspective of metacognition [30]. Here, metacognition is separated into two separate components: Knowledge about cognition and regulation of cognition [31]. Knowledge about cognition includes declarative knowledge, as well as procedural and conditional knowledge (what, how, when, and why that incorporate problem solving, reasoning, and planning), and the component regulation of cognition includes self-regulation. Self-regulation is here seen as a combination of emotion and cognition, i.e., deciding when to start, when to continue, and when to stop an action in order to achieve a goal. Here, the individual chooses his/her behavior, plans a response, and selects the intensity of the response [31].

### 1.3. Different Types of Skills Supporting the Developmental Perspective of Executive Functions

The perspective chosen here is the developmental perspective of executive functions. EFs develop rapidly and individually, especially for children of ages 3–7 years old [32]. For very young children, the initial development is a change from the need of immediate satisfaction to more intentional actions, a process that includes moving from external to internal regulation [33]. An example of a more detailed developmental path has been suggested by Kopp (1982), [34] who described a general developmental trajectory for young children (about 12 months of age to about 36 months of age) where the child moves from noticing social demands and being able to inhibit certain behaviors in response to external influences, toward delaying behavior on request and regulating behavior in the absence of external monitors with a flexibility to meet changing demands.

Others have suggested more specific trajectories or sets of skills for the individual components, especially for IC. For IC, important factors are inhibiting the impulse to respond by waiting and thereby maybe providing a more conscious response or delaying a reward waiting for a greater reward [35], focusing on the task at hand and being able to sustain that attention. At the same time, more emotional skills have also been defined, such as empathy, cooperation, and role-coordination, which include recognizing and labeling one’s own and others’ emotions and modulating one’s own emotions, as well as controlling behavior. Within this approach, problem-solving skills are not separated from regulation skills, but are instead labeled social problem-solving skills, which include such processes as being able to access problems, consider different perspectives, generate solutions, and negotiate the plan of action with others. Of course, these skills also vary with regard to motivation; regulating one’s attention when the task at hand is less motivating, “cold situations” take more of an effort than regulating one’s behavior in more motivating circumstances, “hot situations.”

### 1.4. Research on Improving EF Skills

Different attempts to support the development of EFs have been considered by researchers. Bierman and Torres (2016), [21] reviewed the research efforts concerning the development of children’s EF skills and found five different types of approaches used in interventions. The first category was direct training of EF skills such as training working memory [36] or attention control [37]. The second category involved a focus on teacher–child interactions and positive classroom climates [38]. The third category was using play as a context for EF interventions, the fourth category was promoting social-emotional learning, and the final category was labeled other promising approaches [21], such as parental interventions and physical activity.

Play is important for children’s natural development to occur in “hot situations” (situations that are motivating). Play can involve teacher-designed, play-based learning activities, play world, or socio-dramatic play [22], where creating and maintaining the context for play uses WM, and assigning characters and their different roles requires adjustment to others while making use of representations. IC, persistence, problem solving, and AS are natural parts of adjusting to characters and changing scenarios. EFs can also develop in activities with less planned focus on EFs, such as having the child adapt to the narrative of the play during free time and social play. Nonetheless, EFs are viewed as highly relevant to learning and having a positive, domain-general effect. Indeed, teacher–child, adult–child, or older peers–child interactions are fundamental for the development of EFs, and in formal settings, it is essential to create an environment that is supportive of emotional and social development by being predictable and encouraging, while preventing behavioral problems.

All the above research has one thing in common: The recognition of an active social learning environment for developing EFs. Some examples of activities used in a preschool context are robotics for empowering teamwork and problem-solving skills [39]. Other hands-on experience has also been suggested, but with the focus to help transform abstract concepts into more concrete concepts [40]. Here, science activities were analyzed as a means to bridge this gap between imaginative and everyday encounters.

The aim of the present paper is to describe the progression of EFs during a longitudinal study of science activities.

## 2. Materials and Methods

### 2.1. Study Design

The study was initially designed for exploring children’s emerging chemistry through a series of science activities. Twenty children from two preschools in Sweden partook in the study, and they were about three years of age at the beginning of the project. The project spanned in total over fifteen months. The intervention was designed in the form of conceptual play [41], which is a play-based learning approach that embeds scientific concepts within the child’s everyday activities at preschool. Here, the preschool teacher takes an active role in supporting the child during activities, and as such, the teacher was an important part of all activities during the course of the study. This play pedagogy is known as sustained shared thinking [42], and it requires that the teacher and children actively work together.

The overall design of the activities was dynamic in nature and followed those of an educational experiment [43], where data from the activities were analyzed immediately after completion and the next session was designed based on this analysis. This design method made it possible for the progression of content to match the children’s needs, i.e., to further the emergence of a scientific content [44]. The overall goals of the activities were initially designed to encourage the children to take their imagination beyond the visual level toward an invisible level and to explore how the children would perceive this invisible level (see Table 1).

**Figure 1:**
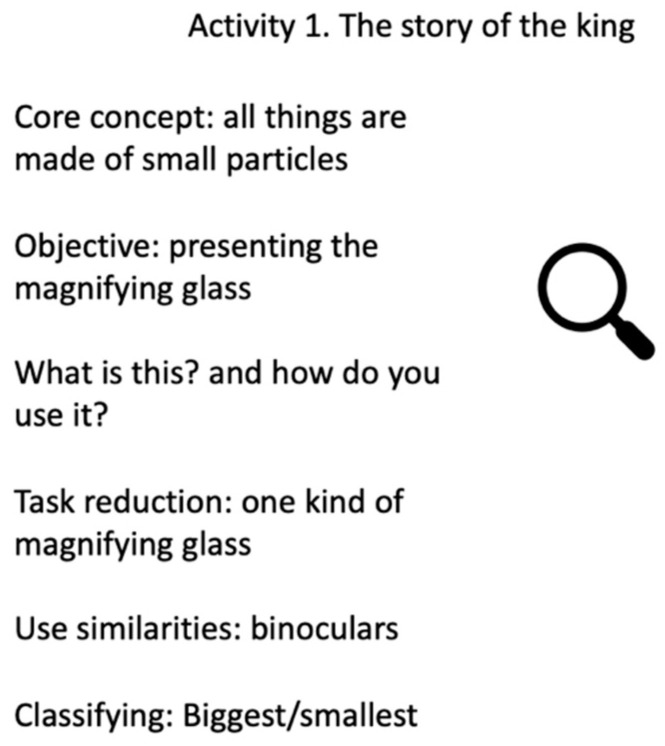
The general outline of activity 1, which included the story of the king and his birthday.

Despite all efforts, it was only when the children were provided with visual experiences of the transition between the macro and the sub-microscopic level through zooming-in videos that the children were able to include experience of the sub-microscopic level into their imagination [45]. The activities were designed with the king and his family as a backdrop. Initially, the king had just had his birthday and had received a magnifying glass as a birthday present. In the following activities, the focus became exploring the magnifying glass and items in the children’s immediate surroundings at preschool. The general outlines of the activities were summarized in scripts such as the one in Figure 1.

The study was approved by the regional ethics committee, Lindöping, (Dnr 2018/451-31). As visual ethnography requires immersion in the context of the study [46], the researcher took the teacher’s role during the intervention. The researcher was a doctoral student that was qualified as a preschool teacher and held a master’s degree in developmental psychology.

Visual ethnography enables the researcher to be immersed in the data collection as an active participant. The method provides means for collecting rich data, as the researcher has the opportunity to create situations and ask questions to explore the situation at hand. By recording all activities, the researcher can revisit data and also include body language into the analysis, something that is essential when working with young children. Being immersed in the data collection in this manner also presents a risk for bias in the analysis; an effort to avoid this bias was made by having three different researchers initially analyze the data separately and then compare their individual analyses.

### 2.2. Data Analysis

All of the recorded sequences were included in the data analysis, and they were examined for indicators of development of the three major parts of EFs: WM (working memory), IC (inhibitory control), and (AS) attention shifting. General indicators of the three components (WM, IC, and AS) were derived from either body language or verbal cues, and the activities were seen as dynamic situations where each child could either inhibit the surroundings (including the other children) or inhibit the planned activity. 

In this first analysis, WM was found in all the sequences involving children from three years of age and onward. In each of the activities, a task related to the background story of the king was given to the children. The children remembered the given task on all occasions, a result that suggests that for these children, WM had already developed enough to keep the backdrop of the activity in mind. For IC and AS, we derived a series of general indicators based on the children’s verbal expressions and body language (see Table 1).

**Figure 2:**
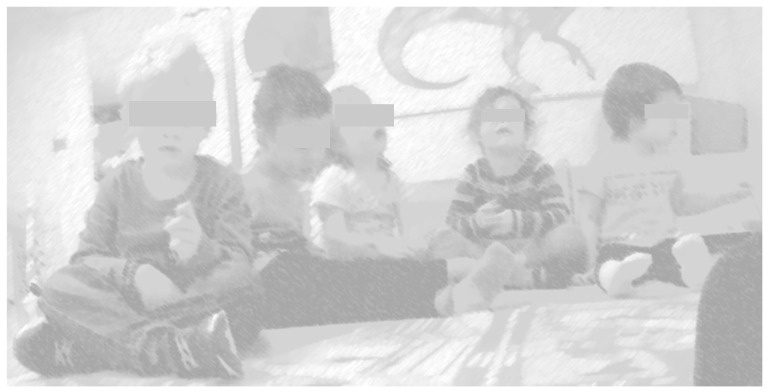
Body language in one instance of the first activity.

For IC, the body language expressed by the children could only be analyzed for its progression. No conclusion could be drawn regarding if the body language ICs were either intentional inhibitions or a lack of inhibitory control. Nonetheless, the general indicators for IC included body language where children shifted their focus from the ongoing activity toward items in their immediate surroundings or to their own person by moving around or standing up (see Figure 2). Another indicator was acting out, something that resulted in the other children shifting focus from the activity at hand. Additional indicators of body language for IC (see Table 2) were sitting down and maintaining eye contact with the teacher or moving very close to the teacher to look at the objects for the activities. Huddling over the objects did, in fact, inhibit everything but the activity.

The verbal inhibitions concerning either the activities or the surroundings were more easily analyzed with regard to intentions. One example of this type of inhibition was contributing with one’s own experiences that had no connection to the present activity; “my grandmother’s name is Sibyl,” which was interpreted as an intentional inhibition of the activity.

Indicators for AS (attention shifting or change of perspective) included acknowledging other perspectives, taking another’s perspective here by imagining being one of the role characters in the story of the king, feeling sorry for the king when he was ill, and creating new or additional narratives with other children during the activities. Data included here and the change described occurred over a time period of 10 months when the children (youngest to oldest) ranged between the ages of 3 years old to 3 years and 10 months at the beginning of the study and 3.5 years to 4 years and 4 months at the conclusion. The group consisted of 8 girls and 12 boys.

## 3. Results

In the analysis, at first, AS and IC seemed to be discernable, but as the activities progressed, they merged into verbal social interactions where inhibitory control naturally evolved and allowed the children not only to inhibit the surroundings (their friends), but also to make micro-inhibitions of the science activity in favor of creating different or alternative social narratives.

In the first activity, when the story of the king and the royal family was introduced, the children showed some of the indicators of IC. Some children sat down and listened with their eyes completely focused on the teacher, while others showed other signs of inhibition, turning their focus toward a set of pens in the vicinity of where the activity took place, as well as toward the mat on which they were sitting (see Figure 2).

### 3.1. The First Set of Activities

The conversation for the first set of activities (ranging in time over 1 month) was mainly directed toward the teacher, even though sometimes the comments that the children themselves contributed with were made as responses toward other children’s comments. The conversation involved mainly statements concerning the children’s own perspective, such as statements of what they themselves preferred. The king’s birthday cake was a topic that arose in several activities, and the children’s conversation concerned mainly which flavor of birthday cake they themselves preferred; “I like chocolate,” “I like strawberry and rainbow,” “I don’t like cakes.” In one of the activities, one of the children was sick, and another child commented on the fact by stating that “Wilhelm is sick.” The response from another child was “did you not first see that I was here,” a statement that was not further expanded upon. For this first set of activities, IC was not visible.

The indicators for AS were also initially few as the children mainly made statements regarding their own perspectives. The first sign of AS within this set of activities (activity, five) was found when the children began playing “the royal family”; one example of this was when two boys had decided to be a knight and a prince, and they immediately began sword fighting, making sound effects, but without a verbalized plan for the play, other than “I will catch a dragon for you.”

**Figure 3:**
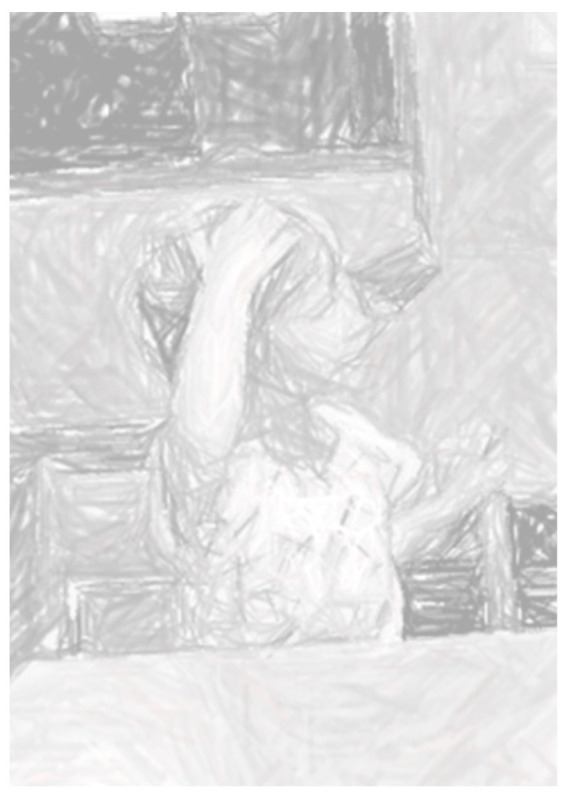
One of the children showing a change of perspective, feeling sorry for the king, holding her head stating that “he was sick, poor king.”

Another sign of a change of perspective emerged when the children were recollecting the session when the king was sick. Here, the children held their hand on their head, indicating either that their head hurt or that they had a fever, together with comments regarding “the poor king” (see Figure 3).

At the end of the first month, the first verbal indicator of AS toward another child’s perspective was found. This came as a response to a birthday cake scenario when one of the children stated that if we are going to make a cake, “then William can’t be here, he doesn’t like cakes.”

As the activities progressed and language skills advanced, the children began to refrain from the need to move physically closer to the teacher when the objects used in activities were introduced, although their interest was very high. Five months into data collection, social interactions began to develop. One example of the first of these interactions was derived from one of the final activities before summer break. The children smelled vinegar, a smell that all agreed was disgusting. This common agreement led to the children pretending to fall on the floor being sick or dying, while making noises as if they were throwing up.

Now, the children’s verbal contributions also began taking on a different form. When one child verbalized something, the others did not immediately respond with their own version of the statement; instead, they listened. This was the beginning of one of the first alternative stories that the children began to develop. The following example is derived from one of the recollections of the previous activities where the king was sick and could only lay in bed and listen and smell what was going on in the castle: “There were knights and there was cooking and then you listen and hear something, and then you tell the guards or the knights that the king was sick.” The elaborations of the storyline included telling all the knights and guards that the king was sick; these were details that the other children were quite eager to hear. From this point on, the choice of prioritizing between the activity and other social interactions also became an option.

In the first session after summer holidays (children ages 3 years and 10 months to 4 years and 3 months), more alternative stories began to develop. In the first session, the children sat down, and the teacher only had time to say “good morning” before the first alternative storyline was developed. One of the children had a cuddly cat with her in the room, and the activity began with feeding the cat as it was hungry and could not wait. Discussions were no longer entirely competitive, and micro-inhibitions of the present activity were created as alternative storylines became a frequent part of all activities. At this point in time, the children did use multiple perspectives as they expanded upon other ideas and shifted their attention between different aspects of the activities. This development of AS was, indeed, not always favorable for the science content but did provide a bridge for transferring their use of executive functions, from fairy tales and games toward everyday tasks.

### 3.2. Summary

For these children, their AS developed from displaying their own perspective to recognizing others’ perspectives, a development that was first visible through the fictive story of the king and his knights. Making the shift toward other children’s perspectives was a more difficult task and was initially visible through recognizing another child’s dislike for cakes. A rapid change had begun to emerge just before summer holidays when the children were listening to each other without stating their own opinions, and additional storylines had begun to develop where the children adapted different perspectives and increased their options to include social interactions as a new way to inhibit the planned activities.

Development of IC for these children changed from inhibiting the surroundings or the activity by focusing on themselves or items in their immediate surroundings, toward a more collective inhibition by social interactions.

## 4. Discussion

Analysis of the data from the perspective of EFs provided an opportunity to explore the progression of EFs in a setting that was not designed for this purpose and to explore the consequences of this development on planned activities. Results from this data analysis support the conclusion made by [32], who described the development of EFs as rapidly progressing during the ages of 3–7. One of the reasons for analyzing EFs is that the development of EFs is seen as one important part of school readiness [20]. IC is important for the child to be able to focus on lesson content, by blocking out interferences. At the end of the study, these children did show IC, but instead of blocking out their peers, the social interplay the children chose partially blocked out the activity. From this perspective, the development of CF and IC for the children included in this study did in fact not support school readiness. However, from the perspective of child development, partaking in social interplay is important, and it may be more important for children at this age than focusing on content. It could not be determined from this data set whether the children displayed IC as intentional actions or as a nonintentional action. Therefore, only inhibitions were analyzed, and from this perspective, inhibitions of the science activities increased as CF developed.

When looking from the perspective of preschool and science activities, the micro storylines that developed were sometimes connected to a peripheral aspect of the activity and, sometimes, they were seemingly irrelevant for the activity, but apparently relevant from the children’s perspective. Nonetheless, this transition was the way that these children began to move away from the initial storyline that was introduced as a backdrop to the activity toward making their own connections with aspects relevant to them. This transition placed the science activity in their everyday world, and this event shows the possibilities of using science activities as a way to connect the fictional world with the living world. Such a transition has been shown to be difficult [40].

The children within this study first changed their individual perspective and adapted to fictive figures before they changed their perspective in favor of the other children’s perspectives within the group. This could simply have been a matter of choice, as it may have been more appealing to be a knight or a princess rather than adapting a perspective toward reality. This finding shows the importance of teachers helping children make the connections between the different worlds and supporting the transfer of executive functions from fiction to reality.

The development described here is only the beginning of the development toward school readiness, and the children here did not yet display the ability to inhibit the other children’s invitations, such as when alternative narratives were offered.

One of the limitations to this approach is that the design was not adapted to the in-depth exploration of all aspects, such as the development of WM.

## 5. Conclusions

Results show the importance of science activities within a preschool setting not only for the development of science itself but also for supporting the development of children’s AS and IC, which is a basis for their social development. The teacher has an important role as a narrator, and the shift between the fictive and the real world in play-based science activities can be a bridge transferring EFs from fairytales and fiction to the real world.

## Figures and Tables

**Table 1:** A summary and timeline of the activities included into the study.

**Stage 1**	**Activity Number**	**Content of Session**
250118	1	The story of the king
300118	2	The magnifying glass
010218	3	Close-up videos
060218	4	The magnifying glass
080218	5	Where did the sugar go?
130218	6	Dissolving
150218	7	Filtering
080518	8	Baking
150518	9	Touching
220518	10	Smelling
300518	11	Hearing
310518	12	Disassembly of chocolate ball
070618	13	Recollecting
**Summer Break**		
**Stage 2**	**Activity Number**	**Content of Session**
151018	14	Disassembly of Lego
181018	15	Disassembly of salt and sugar
231018	16	Disassembly of bath bomb
251018	17	Zooming-in video
080119	18	Zooming-in video
100119	19	Zooming-in video
150119	20	Disassembly of sugar
170119	21	Changes of state
190319	22	States of matter
200319	23	Zooming-in videos
090419	24	Mixing

**Table 2:** A summary of the indicators for inhibitory control.

IC (Body Language)
Shifting focus to the surroundings (picking up items)
Moving around (jumping, standing, dancing) Moving close to the teacher
Eye contact with the teacher and little movement
